# From variants to answers: The evolution of genetic counseling in IEI

**DOI:** 10.70962/jhi.20250211

**Published:** 2026-03-20

**Authors:** Blanca García-Solís, Rebeca Pérez de Diego, Silvia Sánchez-Ramón

**Affiliations:** 1 Department of Clinical Immunology, Institute of Laboratory Medicine and IdISSC, Hospital Clínico San Carlos, Madrid, Spain; 2 Interdepartmental Group of Immunodeficiencies, Madrid, Spain; 3 Laboratory of Immunogenetics of Human Diseases, IdiPAZ Institute for Health Research, La Paz University Hospital, Madrid, Spain; 4 Innate Immunity Group, IdiPAZ Institute for Health Research, La Paz University Hospital, Madrid, Spain; 5 IdiPAZ Institute for Health Research, La Paz University Hospital, Madrid, Spain

## Abstract

Inborn errors of immunity (IEIs) comprise >500 rare congenital disorders of the immune system, characterized by susceptibility to infection and immune dysregulation. Genetic testing advances have improved the comprehension of their molecular mechanisms and informed personalized therapeutic strategies. Nevertheless, the interpretation of variants and their clinical relevance remain challenging. Together with the technological limitations of next-generation sequencing and emerging methods, this highlights the need for standardized, reproducible approaches. The decision-making needs to incorporate effective genetic counseling, ethical and communicative considerations, and collaboration between clinicians, geneticists, and bioinformaticians. Ensuring equitable access to advanced genetic diagnostics is crucial to support accurate diagnoses, guide clinical management, and inform family planning. All this together highlights the need to combine clinical expertise and genetic research into an interdisciplinary collaboration, enabling individualized treatment and improved outcomes for individuals with IEIs.

## Teaser

Garcia-Solis et al. examine genetic testing advances in inborn errors of immunity, highlighting challenges in variant interpretation and the need for standardized approaches. The work emphasizes integrating clinical expertise with genetic research through interdisciplinary collaboration to enable personalized treatment, equitable diagnostic access, and improved outcomes for affected individuals.

## Introduction

Inborn errors of immunity (IEIs) (also known as primary immunodeficiencies) are an expanding heterogeneous group of >500 congenital genetic defects. Although classically described as monogenic and germline, immune dysfunction can also result from somatic mutations and other genetic mechanisms beyond this traditional framework, ultimately leading to impaired immune function (1, 2, 3, 4).

High-throughput genetic analysis, such as next-generation sequencing (NGS), has become a critical tool to address the heterogeneity and overlapping phenotypes of these disorders. Although many clinical decisions are based on the patient’s immunobiology and clinical phenotype, genetic testing remains highly valuable not only for the diagnosis, clinical follow-up, and treatment optimization of IEI patients, but also for reproductive counseling. Beyond diagnosis, genetic results inform prognosis and guide therapeutic decisions, including the use of targeted treatments or hematopoietic stem cell transplantation (5, 6, 7).

Individual variability makes IEI diagnosis challenging, leading to delays that can be harmful or even fatal (8, 9). Even though 21% of the IEIs are described initially in individual patients (10), presenting *de novo* mutations or the first appearance of an autosomal recessive disease (2), family history is relevant, though incomplete penetrance can complicate pedigree analysis. Identifying causative mutations not only enhances biological knowledge, accelerates future diagnoses and patients’ outcomes (11), but also enables family counseling and preventive strategies for at-risk relatives.

## Genetic testing technologies for IEI: Current strategy and limitations

New genetic sequencing technologies like NGS, whole-exome sequencing (WES), and whole-genome sequencing (WGS) have dramatically improved the identification of single-gene birth defects causing immune system disorders compared with previous methods, such as Sanger sequencing and targeted gene panels (TGPs) (10). The key advances are the ability to interrogate hundreds to thousands of genes simultaneously, increased sensitivity for diverse variant types, and rapid turnaround times.

**Figure 1.**
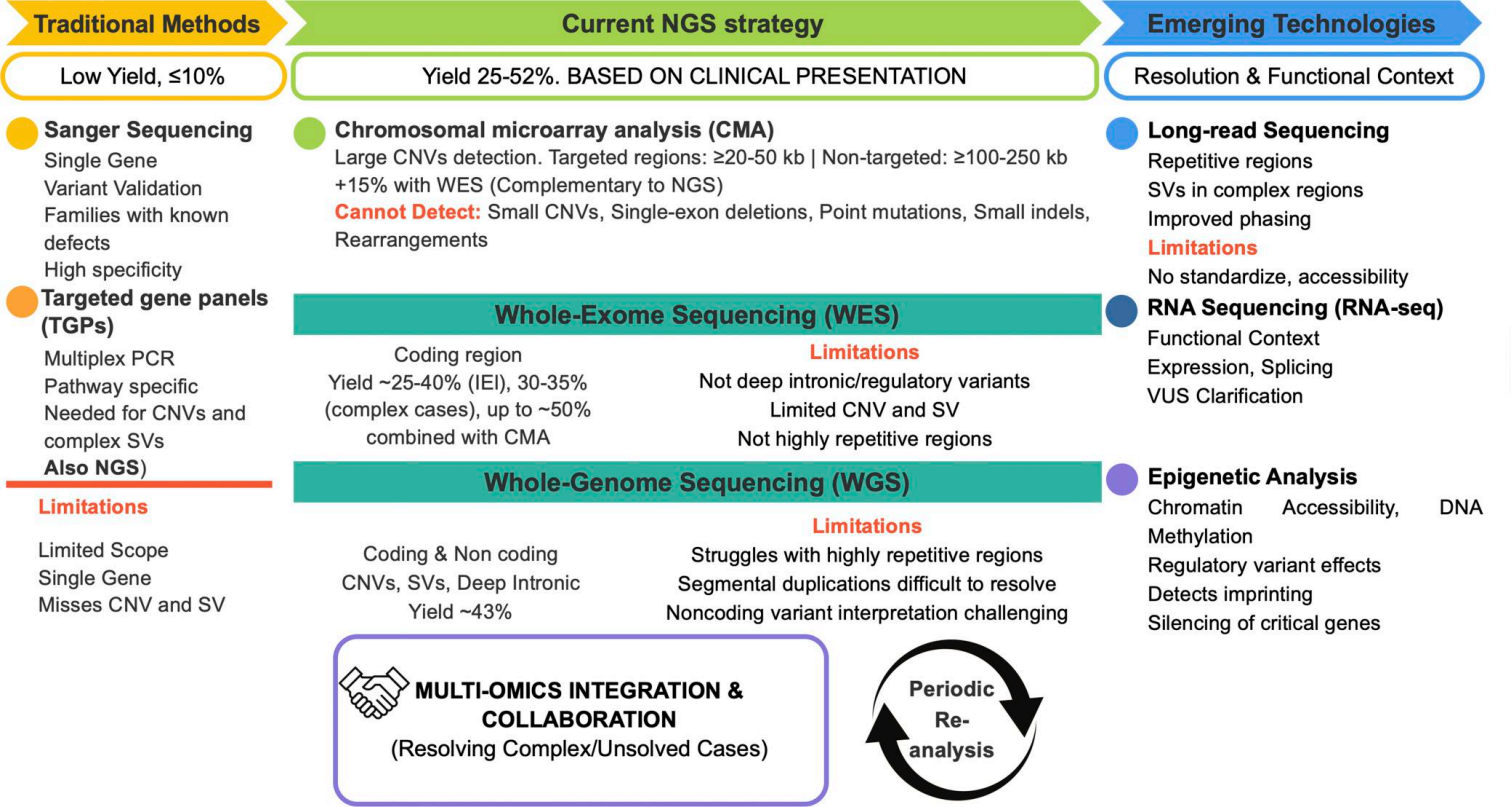
**Summary of the evolution of genetic techniques used for IEI diagnosis.** CNV, copy-number variant; NGS: next-generation sequencing; SVs: structural variants; TGPs: targeted gene panels; VUS: variants of uncertain significance; WES: whole-exome sequencing; WGS: whole-genome sequencing.

Current genetic testing approaches for IEI utilize a tiered strategy based on clinical presentation and suspected genetic architecture (Fig. 1 and Table 1).

**Table 1.** Summary of genetic techniques used for IEI diagnosis. CNV: copy-number variant; NGS: next-generation sequencing; SVs: structural variants; TGPs: targeted gene panels; VUS: variants of uncertain significance; WES: whole-exome sequencing; WGS: whole-genome sequencing

Technique	Function	Key capabilities	Limitations	Diagnostic yield	Best use
Sanger sequencing	Targeted single-gene sequencing	Variant validation; known mutations; family screening for known defects	One gene at a time; cannot detect large deletions/duplications	Validation tool	Confirming specific variants, family cascade testing, and validation of NGS findings
TGPs	Pathway-specific multigene analysis	Cost-effective for specific pathways; faster than WES/WGS; good depth of coverage	Limited to preselected genes; lower yield than WES/WGS	10–25%	Known pathway abnormalities; initial screening
CMA	Large CNV detection	Detects large CNVs (≥20–50 kb targeted, ≥100–250 kb nontargeted); identifies aneuploidies; detects microdeletions/microduplications; unbalanced rearrangements	Cannot detect small CNVs (<10–20 kb); no balanced rearrangements; no point mutation detection	+15% when combined with WES	Syndromic presentation; complementary to WES
WES	Coding regions	Analyzes ∼20,000 genes simultaneously; detects SNVs and small indels; enables gene discovery; periodic reanalysis possible; high sensitivity for coding variants	No intronic/regulatory variants; limited CNV detection; poor coverage of repetitive regions; VUS interpretation challenges	25–40% (IEI cases); 30–35% (complex cases); 36–51% (critically ill neonates); up to 50% with CMA	Complex/unknown IEI; negative targeted panel; phenotypically diverse presentation; gene discovery
WGS—short-read sequencing	Comprehensive genome-wide analysis	Detects coding, intronic, and regulatory variants; better CNV and SV detection than WES	Expensive; complex data interpretation; struggles with highly repetitive regions; large VUS burden	43%; superior to WES for undiagnosed cases	WES-negative cases; regulatory variants; complex SVs
Long-read sequencing (WGS)	Ultra-long-read genome analysis	Superior repeat expansion detection; resolves highly repetitive regions; better SV detection; identifies variants in previously inaccessible regions	Not yet standard in clinical practice; higher cost; limited clinical validation; requires specialized expertise; lower throughput than short-read sequencing	Better than short-read WGS; identifies novel variants missed by other methods	Short-read negative cases; suspected expansions; complex SVs; research applications
RNA-seq	Transcriptome analysis	Validates splice-site variant effects; detects aberrant splicing; identifies allelic imbalance and exon skipping; measures gene expression levels; reveals monoallelic expression	Tissue-specific expression patterns; cannot detect all DNA variants; temporal expression variability	Resolves ∼35% of VUS cases; functional validation tool	Validating variants; explaining splicing defects; complementing DNA sequencing

**Single-gene Sanger sequencing** remains the gold standard for validating variants and for targeted testing in families with known defects, offering high specificity but limited scope (12).

TGPs are employed for pathway-specific disorders, providing focused analysis with diagnostic yields generally lower than exome/genome sequencing (13). Not all TGPs utilize NGS technology; some employ alternative methods such as Sanger sequencing, multiplex PCR, or microarray-based capture, especially for small panels or specific variant types. Most current TGPs use NGS, but ancillary methods may be required for certain copy-number variants (CNVs) or complex structural variants (SVs) (14).

**Chromosomal microarray analysis (CMA)** is primarily used to detect CNVs in syndromic phenotypes and can increase diagnostic yield by up to 15% when combined with exome sequencing, particularly in pediatric cohorts with complex presentations (7, 15). CMA can detect CNVs such as large deletions, duplications, microdeletions, and microduplications typically >20–50 kb in targeted regions and 100–250 kb in nontargeted regions. CMA is effective for identifying aneuploidies, unbalanced rearrangements, and some forms of mosaicism, but its sensitivity for mosaicism is platform-dependent and generally limited (7).

Nevertheless, CMA cannot reliably detect small CNVs below its resolution threshold (often <10–20 kb), single-exon deletions/duplications, small indels, point mutations, balanced rearrangements (e.g., translocations, inversions), or CNVs in regions not represented on the array platform (7).

### Whole-exome and whole-genome sequencing

Monogenic diagnoses are achieved in ∼25–40% of patients with classic IEI phenotypes using targeted approaches (WES/WGS), and up to 50% when combining with CNV or CMA (16, 17, 18, 19). The rapidly evolving field necessitates periodic data reanalysis and integration of clinical practice with basic research, as new gene–disease associations and improved bioinformatics tools continue to emerge. Reanalysis of exome data can yield additional diagnoses, especially as novel disease genes are discovered and variant interpretation frameworks are refined (3, 20).

While WGS improves detection of CNVs, repeat expansions, and complex rearrangements compared with WES and CMA, limitations remain, especially for highly repetitive regions, segmental duplications, and certain insertions. Short-read WGS may miss SVs in these contexts, and long-read sequencing offers superior sensitivity for repeat expansions and insertions but is not yet standard in clinical practice (15, 16, 21). While bioinformatics tools are improving CNV detection from NGS data, their clinical validation is ongoing (7), and expert interpretation alongside functional studies remains essential for accurate diagnosis and assessment of variant pathogenicity.

WGS enables the detection of deep intronic and regulatory variants, which are missed by WES and TGPs. However, interpretation of noncoding variants is challenging due to limited functional annotation and predictive tools, and their clinical significance often remains uncertain (22, 23, 24).

NGS-based approaches achieve diagnostic yields of 25–52% in suspected IEI, compared with 10% or less with traditional methods (25). Clinical exome sequencing has provided molecular diagnoses in ∼30–35% of complex cases, with actionable findings in most diagnosed patients, and achieves 36–51% diagnostic rates in critically ill neonates with direct clinical impact (18, 25).

WGS detects SVs, copy-number changes, deep intronic mutations, and repeat expansions missed by WES, achieving diagnostic yields of 43 versus 10% for standard testing, identifying causal variants in patients who were undiagnosed by WES (24). Long-read sequencing further improves detection of previously inaccessible regions and has enabled identification of novel IEI-causing variants, such as SASH3 and IKBKG defects, undetectable by conventional methods (26).

Because of all of that, collaboration between clinical and research teams and the use of multiomics approaches are increasingly important for resolving complex or unsolved cases (27).

### Emerging technologies

**Long-read sequencing platforms**, such as Pacific Biosciences (PacBio) and Oxford Nanopore Technologies (ONT), produce reads spanning tens to hundreds of kilobases, enabling resolution of repetitive regions, SVs, and complex rearrangements that challenge short-read methods. While 50–69% of the human genome is repetitive and causes short reads to be unmappable or multimapping, long reads anchor in unique flanking sequences to resolve these ambiguities (28, 29). Recent improvements have achieved >99.9% per-base accuracy for PacBio HiFi and comparable ONT Duplex performance, with SNV detection rivaling Illumina in complex regions (30, 31). However, small indel calling, especially in homopolymers and tandem repeats, remains more challenging for ONT, though ongoing improvements in base calling algorithms and quality control tools (e.g., PEPPER-Margin-DeepVariant) have further reduced artifacts and improved variant calling performance (32, 33, 34). Long-read sequencing enables megabase-scale variant phasing, direct detection of DNA methylation and epigenetic modifications, haplotype-resolved assemblies, and parent-of-origin analysis—capabilities absent in short-read methods (34). More accurate genome assembly with long-read sequencing benefits individuals from diverse ancestries by enabling the construction of high-quality, haplotype-resolved, and near-complete diploid genomes, overcoming the limitations of current reference genomes that are incomplete and not representative of global genetic diversity. This is critical for capturing population-specific SVs and for building pangenome references that better reflect human diversity (35).

RNA-sequencing (RNA-seq) and epigenomic techniques can enhance the interpretation of genetic variants by providing functional context beyond DNA sequencing alone. RNA-seq enables direct assessment of the impact of variants on gene expression, splicing, and allele-specific expression, which is particularly valuable for clarifying the pathogenicity of variants of uncertain significance (VUS) and for identifying aberrant splicing events that may not be predicted from DNA data alone. RNA-seq is effective for canonical and noncanonical splice-site variants, deep intronic variants, and regulatory variants affecting transcript structure (7, 36), and reveals functional consequences in untranslated regions, promoters, and enhancers through expression or isoform changes (37, 38, 39). Epigenomic assays, such as those measuring chromatin accessibility or DNA methylation, further inform the regulatory landscape and can help prioritize noncoding variants with potential pathogenic effects (40). Integration of these multiomics data types increases diagnostic yield and provides mechanistic insight into disease etiology, especially in cases where DNA sequencing alone is inconclusive.

## Current diagnostic challenges

### Diagnostic yield and technical limitations

Nonetheless, the overall diagnostic yield is below 50% (9, 41, 42), and inconclusive results, such as VUS and genes of uncertain significance (GUS), refer to cases where pathogenic variants are identified in genes lacking established disease associations or sufficient evidence for causality in IEI. These findings necessitate interdisciplinary interpretation and longitudinal follow-up.

Technical limitations, such as incomplete coverage, variant interpretation challenges, and population-specific biases, must be considered, as no single test captures all pathogenic variants (43, 44). Beyond these knowledge challenges, current technical limitations in high-throughput genetic and genomic analysis include the complexity of bioinformatics pipelines and limitations in detecting certain variant types (e.g., repeat expansions, SVs, or regions with pseudogenes or large deletions). Analytical validity and uniformity across sequencing panels remain problematic, and functional validation of candidate variants is often required to confirm pathogenicity (8, 14).

Moreover, gonosomal mosaicism could represent an under-recognized source of phenotypic variability and recurrence risk in apparently *de novo* cases (45). These limitations emphasize the importance of trio-based sequencing (including both biological parents) to improve variant interpretation and refine genetic counseling and reproductive risk estimates.

### Clinical decision-making and test selection

The American Academy of Allergy, Asthma, and Immunology (AAAAI) considers genetic testing an essential tool in evaluating suspected IEI but does not recommend universal high-throughput or population-based genetic screening for all individuals (5, 46). Genetic testing should be prioritized for patients with a clinical suspicion of IEI (such as recurrent infections, immune dysregulation, and lymphopenia) or relevant family history. However, genetic data alone cannot establish a diagnosis. Interpretation of genetic variants requires integration of clinical phenotype, immunologic profiling, population frequency data, functional studies, and family segregation analysis when available. Not all genetic variants are disease-causing, and functional validation is critical as genetic testing alone cannot fully resolve variant pathogenicity or penetrance.

**Figure 2.**
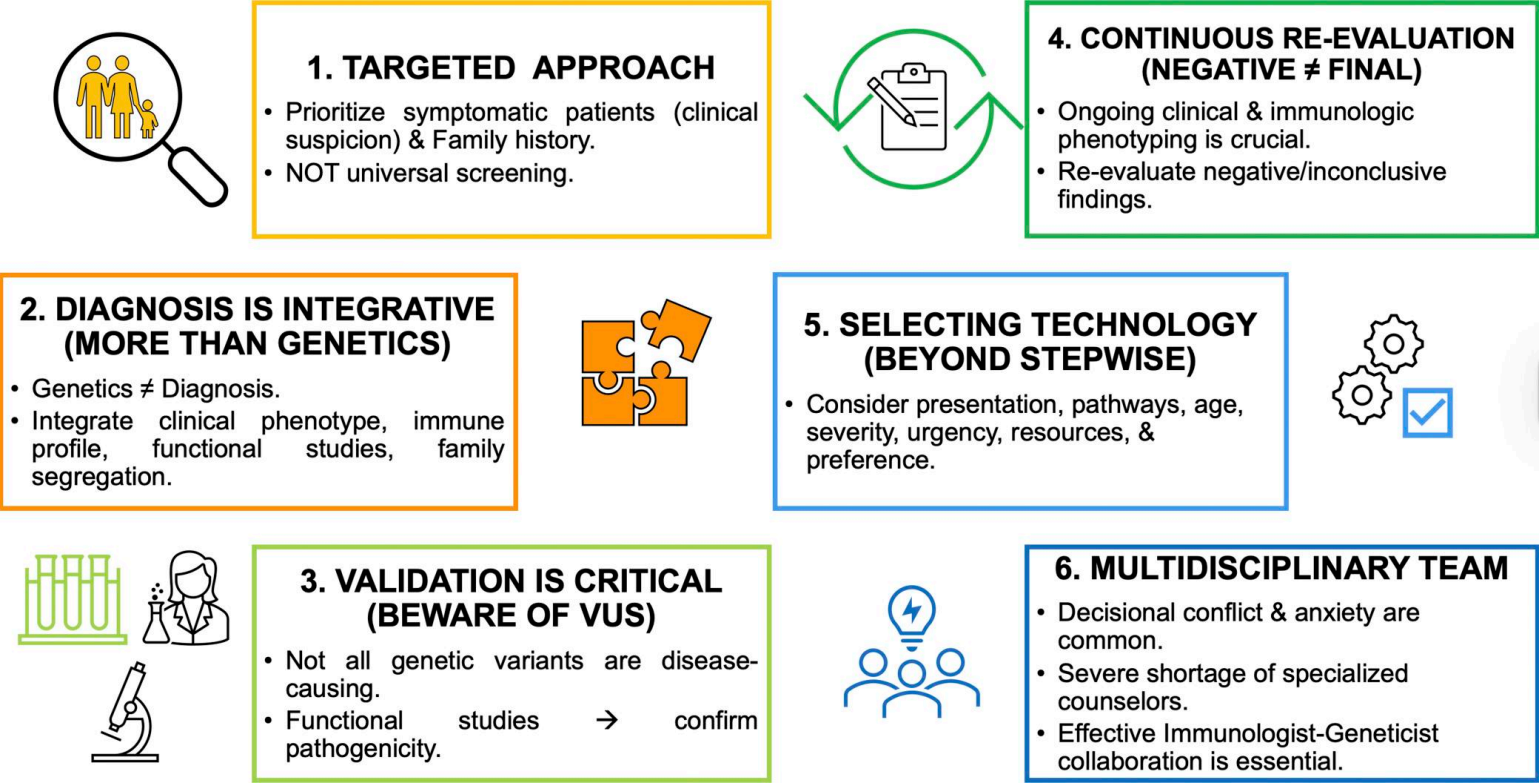
Key considerations in IEI genetic testing.

Ongoing clinical and immunologic phenotyping is crucial regardless of genetic results. Patients with negative or inconclusive genetic findings require continued reevaluation, as evolving manifestations may guide subsequent testing or enable variant reinterpretation. Multidisciplinary expertise is crucial in preventing misdiagnosis (Fig. 2).

Clinicians should approach the selection of genetic technology for suspected IEI by integrating multiple factors beyond the traditional stepwise, phenotype-driven approach recommended by the American College of Medical Genetics (ACMG) (47). Key considerations should include clinical presentation and phenotypic overlap, suspicion of specific affected pathways, age and severity of presentation, family history and inheritance pattern, urgency of diagnosis, institutional resources, cost constraints (even though the cost of sequencing has decreased in recent years (7, 21, 22)), and/or patient preferences regarding incidental findings.

The increasing breadth of available tests can lead to decisional conflict and anxiety. While interdisciplinary collaboration with genetic counselors is recommended, there is a severe shortage of these specialists, and IEI is rarely part of their standard training. Effective collaboration could be challenging; it requires that immunologists and geneticists understand each other’s approaches and limitations, forming a multidisciplinary team.

## Computational tools and variant prediction

In the genomic analysis, there are a lot of VUS and GUS (48), creating a need for computational tools to assist in pathogenicity prediction. While machine learning and artificial intelligence (AI) models show promise (49), their performance is fundamentally constrained by the quality, size, and diversity of training datasets, which are often insufficient for IEI due to disease rarity and clinical heterogeneity.

Current bioinformatics algorithms, including CADD, SIFT, and PolyPhen2, use biochemical and evolutionary data to assess variant harmfulness (7, 8, 15) but were primarily designed for loss-of-function (LoF) predictions. These tools struggle to accurately predict complex mechanisms such as gain-of-function (GoF), dominant-negative, or hypomorphic effects that are common in IEI. Functional validation studies are therefore essential before reaching definitive clinical conclusions, enabling more precise diagnosis and appropriate therapeutic intervention.

Building collaborative databases, having AI as support, to identify genotype–phenotype correlations, represents an important future direction.

## Variant interpretation and clinical relevance

Traditionally, many IEIs have been considered monogenic diseases, but cases of oligogenic and polygenic inheritance have been identified, particularly in common variable immunodeficiency (CVID) (50). However, especially in adult patients, it is becoming evident that we may be dealing with oligo- or polygenic diseases influenced by mutations in multiple genes, then modulated by epigenetics, associations with the major histocompatibility complex, and polygenic interactions (51, 52). It is still too early in the field for establishing scoring systems or tools to be reliably used in clinical practice for assessing the contribution of multigenic variants in IEI. Current variant interpretation guidelines and scoring systems focus on single-gene causality, and the assessment of oligogenic or multigenic contributions remains largely research-based and requires significant clinical expertise and judgment, rather than automated or standardized scoring (7).

The clinical heterogeneity observed in IEI is driven mainly by a complex interplay of genetic, epigenetic, and environmental factors (53). For example, gene modifiers such as TACI (TNFRSF13B) in CVID can modulate disease severity and phenotype, with certain variants acting as risk alleles or influencing penetrance and expressivity (54). In addition, comutations or digenic interactions, for instance, concurrent mutations in JAK3 and CTLA4 or the digenic autoimmune-mediated diabetes syndrome caused by pathogenic variants in both ALDH2 and ADH5, can result in more severe or atypical phenotypes than single-gene defects, reflecting epistatic effects and nonlinear interactions within immune pathways (55, 56). Lastly, autosomal random monoallelic expression (aRMAE) has recently been shown to underlie variable expressivity and incomplete penetrance in dominantly inherited IEIs. aRMAE can result in cell populations expressing either the mutant or wild-type allele, leading to discordant clinical phenotypes even among individuals with the same germline mutation (57).

Environmental and gene-regulatory factors also contribute to heterogeneity. The microbiota can modulate inflammatory and autoimmune manifestations in IEI (36). For instance, in FOXP3- and NOD2-related disorders, dysbiosis influences intestinal inflammation and disease severity. Exposure to environmental insults such as radiation, as in ataxia-telangiectasia, can exacerbate DNA repair defects and contribute to clinical variability. In addition, DNA methylation and chromatin dynamics can modulate gene expression and immune function, contributing to heterogeneity even among patients with identical genetic lesions (58, 59).

Recent efforts, such as the GenIA database (https://www.geniadb.org), are designed to provide users with an up-to-date, integrated view of variants, genes, diseases, patients, and underlying immunobiology, linking these dimensions together (60). This resource is therefore invaluable for comprehensive interpretation and hypothesis generation, but it is not intended or validated as a stand-alone tool for clinical decision-making regarding oligogenic or multigenic inheritance in IEI.

However, oligogenic and polygenic risk scores do exist for systemic autoimmune diseases such as systemic lupus erythematosus and rheumatoid arthritis (61, 62, 63), being refined for risk prediction and stratification, leveraging large-scale genome-wide association studies and extensive patient cohorts. Yet, their application to immunobiologically heterogeneous, genetically undifferentiated cohorts defined only by rheumatologic diagnoses can yield misleading, noncausal associations. These issues, together with oversimplified assumptions about gene–gene interactions, have contributed to the poor or inconsistent performance of polygenic risk scores in immune-mediated diseases.

Defining penetrance and variable expressivity remains challenging, as genes like CTLA4 or PIK3CD have incomplete penetrance (64, 65, 66), meaning some variant carriers may appear unaffected. Altogether, current data highlight the requirement for enhanced bioinformatics (67) and functional validation methods for candidate variants.

## Complex genetic paradigms in IEI

### Balancing LoF and GoF variants

IEI, sometimes, does not fit traditional monogenic disease paradigms. Some genes, such as STAT1, STAT3, CARD11, and PI3KCD, can harbor both GoF and LoF variants, resulting in distinct clinical phenotypes (3). Rather than simple compensation or neutralization, mutations in the same gene or pathway can produce overlapping or paradoxical clinical presentations (7). For example, in the STAT3 pathway, the R335W variant produces a hybrid phenotype with both GoF and LoF features that does not fit classical disease categories (68). Similarly, in other pathways such as SOCS1 and CARD11, different combinations of variants can dysregulate multiple immune pathways, leading to pleiotropic and variable clinical manifestations (69). Somatic mosaicism further complicates this picture: in UBA1 mosaicism, disease severity relates to both the proportion of variant-carrying cells (70) and its presence in nonhematopoietic tissues (71), while in some cases, GoF mutations in a cell subset can partially offset LoF mutations in other cells, resulting in milder phenotypes (72, 73).

These complexities significantly impact clinical decision-making, but genetic findings must be integrated with clinical phenotype and disease severity. Treatment selection, particularly targeted therapies such as JAK inhibitors for STAT1 GoF or PI3K inhibitors for activated PI3K delta syndrome, depends on the patient’s clinical manifestations, not genotype alone. Asymptomatic patients with pathogenic variants may be managed with surveillance rather than immediate intervention, and management strategies must be tailored to the individual patient’s phenotype and disease activity.

Addressing these challenges requires both computational and experimental advances. Genome-wide prediction tools such as LoGoFunc outperform traditional methods by using machine learning with diverse feature sets to distinguish GoF from LoF variants, improving accuracy over traditional pathogenicity predictors (74). Experimental approaches, such as systematic alanine scanning mutagenesis to generate variant catalogs, enable high-throughput functional assessment of variants, as demonstrated for STAT1, where alanine scanning accurately classified LOF and GOF mutations and provided mechanistic insights into disease phenotypes (75).

The integration of AI-driven tools to synthesize genomic, functional, and clinical data represents a future direction for enhancing variant classification accuracy and scalability (76, 77), though these approaches remain limited by dataset quality and diversity in rare diseases.

## Communication and counseling

**Figure 3.**
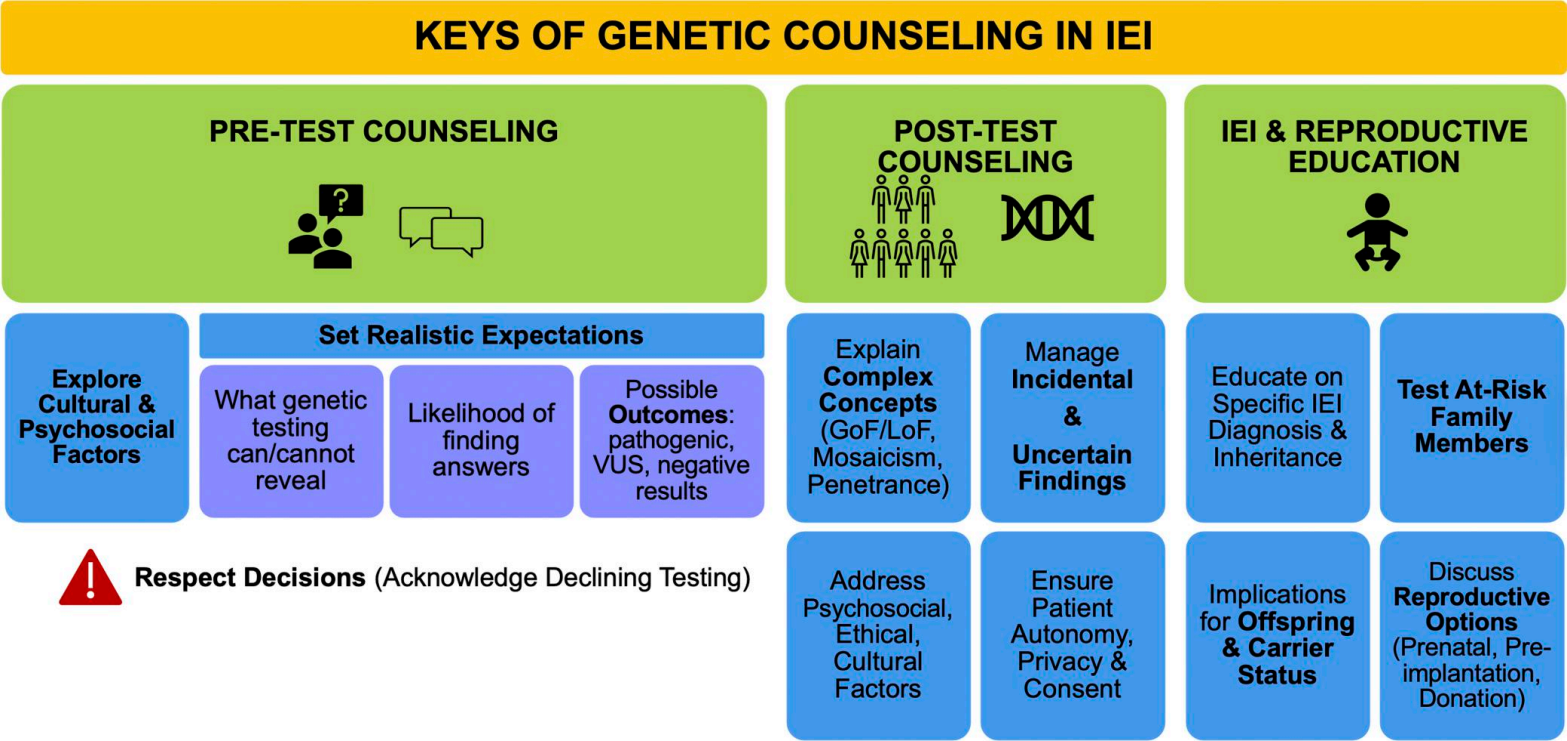
**Summary of communication and counseling.** GoF: gain-of-function; LoF: loss-of-function.

Genetic counseling in IEI requires explaining complex genetic information to patients and their families, adapting to their understanding and cultural background (Fig. 3). Pretest counseling should establish realistic expectations about what genetic testing can and cannot reveal, discuss the possibility of uncertain results, explore cultural and psychosocial factors influencing testing decisions, and acknowledge that some families may decline genetic testing.

Posttest counseling becomes particularly challenging when explaining nuanced concepts such as GoF versus LoF effects, mosaicism, incomplete penetrance, and variable expressivity to families with varying health literacy levels. Counseling must address psychosocial, ethical, and cultural factors that influence reproductive choices, particularly in populations with high rates of consanguinity or specific cultural practices (5, 46). This includes managing incidental or uncertain findings, ensuring patient autonomy, protecting privacy, and addressing concerns about genetic discrimination and data use (12, 78). Because of that, the ACMG highlights the importance of clear consent and privacy protections, especially in the context of carrier and reproductive screening.

Genetic counseling should include education about the specific IEI diagnosis, mode of inheritance, and the implications for offspring. Testing at-risk family members is essential to clarify carrier status and inform reproductive risk (5). The AAAAI recommends it for all affected individuals, carriers, or those at risk, with a focus on inheritance patterns, recurrence risks, and options like preimplantation testing, prenatal diagnosis, and gamete donation to support informed decision-making (7).

### Roles of the clinician and geneticist in the genetic counseling process

Due to the complexity of this matter, collaboration between clinicians (clinical immunologists and pediatricians, but also other specialists and primary care physicians) and geneticists is necessary to seek the middle ground needed to communicate to the patient everything that is relevant without exceeding the limits that they can bear, always prioritizing the patient’s benefit. There is a need for interdisciplinary expertise to interpret results, as most clinicians lack formal training in genomics. There is also incomplete knowledge of gene–disease associations, variable penetrance, and phenotypic heterogeneity, which complicates counseling and management decisions (79). The rapid pace of gene discovery means that reanalysis of data may be necessary as new information emerges.

### How much to inform and when?

While genetic testing is becoming increasingly accessible, the shortage of specialized expertise may elevate the risk of misdiagnosis. Patients need complete counseling before genetic testing about possible inconclusive results, unsolicited findings, and carrier status identification. Results should be delivered by clinicians during specialized consultation, with thorough discussion of medical and social implications to ensure informed decision-making.

The ACMG and the European Society of Human Genetics (80, 81) provide general guidelines for genetic testing, including recommendations for reporting secondary findings in clinical exome and genome sequencing (82).

In the case of VUS, one must be cautious when informing the patient, especially when the information about it is limited. Clinicians must distinguish between variant classification (whether there is sufficient evidence to call a variant disease-causing) and variant interpretation (what it means clinically for the patient’s disease). This could cause psychological distress that, in most cases, is not justified by the severity of the variant. While guidelines recommend VUS should not alter management, some VUS may warrant clinical action despite insufficient evidence for pathogenic classification, and conversely, some pathogenic variants may lack clinical relevance. Providers must assess variants in the context of each patient’s specific phenotype and immunobiology, considering the pros and cons of this information, conducting thorough research on the variant and, whenever possible, performing Sanger verification and functional validation before issuing a genetic report (15, 83).

ACMG guidelines support reporting secondary findings (81, 82), which are intentionally identified pathogenic variants in actionable genes that may be unrelated to the primary testing purpose. Patient consent is required, but clinicians should routinely inform patients about these opportunities and their potential advantages.

## Conclusions and future perspectives

The increased knowledge in genetics and IEIs highlights the need for greater interdisciplinary collaboration between geneticists, immunologists, bioinformaticians, and other clinicians.

As genetic data become more complex, we need to standardize protocols for variant interpretation and validation, ensuring accurate, reproducible diagnoses and clinical settings.

Genetic diagnosis increases the knowledge of molecular mechanisms, improving treatment, determining optimal timing, and identifying new therapeutic targets. It also plays a basic role in family planning and prenatal diagnosis (both pre- and postconception).

Importantly, genetic counseling in IEI should be reframed as a multidisciplinary, patient-centered, and longitudinal process that integrates evolving genomic evidence with immunophenotyping, clinical trajectory, and family values, rather than as a single-point intervention focused solely on molecular diagnosis and risk communication. This perspective acknowledges that variant interpretation in IEI is an ongoing, adaptive process requiring collaboration between genetic counselors and immunology clinicians to revisit recommendations as new information emerges, including variant reclassification, novel therapies, or changes in family circumstances.

Equitable access to advanced genetic testing is essential, and the increasing precision of these technologies enables personalized therapeutic strategies tailored to individual genetic profiles. This integrated approach, combining clinical expertise with genetic research, promises more effective treatments and improved outcomes for IEI patients.

Current approaches in IEI rely on expert-driven, individualized interpretation of genetic findings, often integrating functional genomics and multidisciplinary review, rather than automated risk scoring.

## Data Availability

No new data were generated or analyzed in support of this study.
